# An Improved Pig Counting Algorithm Based on YOLOv5 and DeepSORT Model

**DOI:** 10.3390/s23146309

**Published:** 2023-07-11

**Authors:** Yigui Huang, Deqin Xiao, Junbin Liu, Zhujie Tan, Kejian Liu, Miaobin Chen

**Affiliations:** 1 College of Mathematics Informatics, South China Agricultural University, Guangzhou 510642, China; mingyuzi2022@163.com (Y.H.); morningbear@stu.scau.edu.cn (J.L.); tanzujie97@foxmail.com (Z.T.); m16673413675@163.com (K.L.); cmb13510389173@163.com (M.C.); 2 Key Laboratory of Smart Agricultural Technology in Tropical South China, Ministry of Agriculture and Rural Affairs, Guangzhou 510642, China

**Keywords:** computer vision, object detection, multiobject tracking, pig

## Abstract

Pig counting is an important task in pig sales and breeding supervision. Currently, manual counting is low-efficiency and high-cost and presents challenges in terms of statistical analysis. In response to the difficulties faced in pig part feature detection, the loss of tracking due to rapid movement, and the large counting deviation in pig video tracking and counting research, this paper proposes an improved pig counting algorithm (Mobile Pig Counting Algorithm with YOLOv5xpig and DeepSORTPig (MPC-YD)) based on YOLOv5 + DeepSORT model. The algorithm improves the detection rate of pig body parts by adding two different sizes of SPP networks and using SoftPool instead of MaxPool operations in YOLOv5x. In addition, the algorithm includes a pig reidentification network, a pig-tracking method based on spatial state correction, and a pig counting method based on frame number judgment on the DeepSORT algorithm to improve pig tracking accuracy. Experimental analysis shows that the MPC-YD algorithm achieves an average precision of 99.24% in pig object detection and an accuracy of 85.32% in multitarget pig tracking. In the aisle environment of the slaughterhouse, the MPC-YD algorithm achieves a correlation coefficient (R^2^) of 98.14% in pig counting from video, and it achieves stable pig counting in a breeding environment. The algorithm has a wide range of application prospects.

## 1. Introduction

The number of pigs is crucial information for pig sales and breeding management, and it is extremely important for farmers. Farmers not only need to calculate their own interests based on the number of pigs during the sales process but also need to have a clear understanding of the number of pigs during the breeding process in order to adjust their breeding management plans. However, currently, pig counting is mainly a manual task, which not only has disadvantages such as low efficiency, high cost, and a small counting range, but also can lead to animal welfare issues due to personnel impatience during counting. Accurate pig counting algorithms can ensure that farmers’ interests are not infringed upon in pig sales, improve breeding efficiency, reduce labor costs, and lower the occurrence rate of animal welfare issues [1]. Therefore, pig farmers have a strong demand for a high-precision and high-reliability automatic pig counting method.

In recent years, video image processing methods have gradually shifted from traditional algorithms to deep learning algorithms. Deep learning algorithms, with their high accuracy, faster running speed, and stronger generalization, can be used to solve problems such as image object detection, image instance segmentation, and multiobject tracking from video. Popular image object detection algorithms currently include the faster R-CNN series and YOLO series [2,3,4]. Image instance segmentation algorithms include mask R-CNN, mask scoring R-CNN, and PointINS [5,6,7]; video multiobject tracking algorithms include MHT-DAM, SORT, and DeepSORT [8,9,10]. In the agricultural field, issues such as low efficiency, high cost, low intelligence, and a shortage of labor are becoming increasingly prominent. The emergence of these algorithms provides new ideas for improving agricultural production efficiency [11].

With the development of computer vision technology, an increasing number of computer vision algorithms are being applied in the field of precision agriculture, such as agricultural object detection [12], plant disease and pest recognition [13], animal behavior recognition [14], agricultural object segmentation [15], animal weight measurement [16], and agricultural object tracking [17]. These cases demonstrate the broad application prospects of using computer vision algorithms to solve agricultural problems. Although using computer vision algorithms to solve counting problems is not currently the mainstream research focus, there has been a commercial demand for algorithms to count objects in video images to reduce the manual counting workload over the past few decades [18,19]. For example, Lins et al. [20] designed a method for counting fish based on image density level classification and local regression. Zhao et al. [21] used the DeepLabV3+ network to achieve a recall rate of 91% for counting the number of seeds per silique in long-horned fruit. Gao et al. [22] created an automatic apple counting method using the YOLOv4-tiny detection network and a single-object tracking algorithm. Kestur et al. [23] proposed a target detection network called MangoNet based on deep semantic segmentation, which is used for mango detection and counting. Although these methods for image or video counting are not specifically designed for counting pigs, they provide useful a research foundation and technical solutions for pig counting.

Recently, some scholars have made good progress in the research of pig counting algorithms [24]. For example, Oczak et al. [25] estimated the number of piglets in a pen by extracting three parameters: the number of detected objects, the area, and the perimeter of all objects from segmented images of farrowing sows. Huang et al. [26] proposed a two-stage center clustering network (CClusnet) to solve the problem of partial occlusion during piglet counting. Jensen et al. [27] used a convolutional neural network (CNN) with a single linear output node to estimate the number of pigs in a given area of a pig pen. However, these researchers only counted pigs through images and have not yet focused on the video tracking and counting of moving pigs. Although Chen tracked and counted pigs using a camera mounted on a patrol robot on the roof [28], the number of pigs could only be estimated through the space perception time response filter (STRF), and it was still difficult to effectively solve problems such as pig adhesion, overlap, and occlusion. Therefore, a more accurate, widely applicable, and easily deployable pig counting algorithm that focuses on video tracking is currently lacking. As a popular tracking algorithm, DeepSORT has been successfully applied to counting fish [29], sheep [30], and birds [31]. Although the structures of counting algorithms are very similar, different targets have different motion patterns, appearance characteristics, densities, and shooting angles. Therefore, for application to counting pigs in farming environments, optimization is required based on pig characteristics and the farming environment.

To address the problems of weak applicability, low efficiency, and easy tracking loss in the current process of automatic pig counting, this paper proposes an improved pig counting algorithm based on a YOLOv5 + DeepSORT [32] model, called the Mobile Pig Counting Algorithm with YOLOv5xpig and DeepSORTPig (MPC-YD). To address the difficulty of recognizing local pig body parts, the MPC-YD algorithm includes a YOLOv5xpig object detection network. In addition, the MPC-YD algorithm also introduces a pig reidentification network, a pig tracking method based on spatial state correction, and a pig counting method based on the number of frames in the DeepSORT algorithm. First, the data acquisition and dataset preparation techniques of the algorithm are introduced, and then the structure of the algorithm is described in detail, followed by an analysis of the performance and results of the algorithm.

## 2. Data Collection and Dataset Production

**Figure 1:**
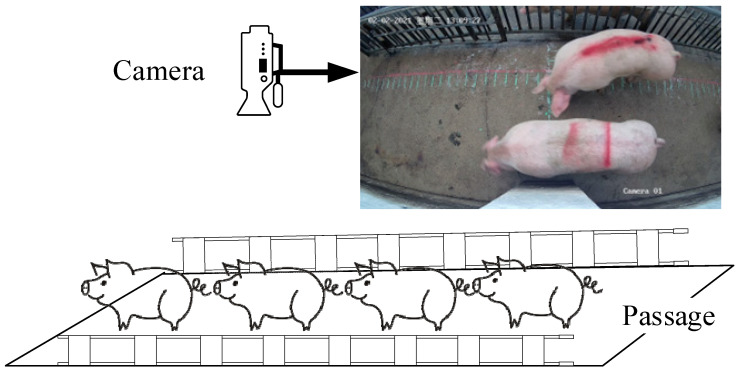
**MPC-YD algorithm** experimental data collection scenario.

The data used in this study were collected from a commercial pig slaughterhouse located in Heyuan city, Guangdong Province, where a total of 1892 pig videos were collected. The experimental data collection scenario is shown in Figure 1. In order to ensure the applicability of the algorithm and animal welfare, the experimental data collection channel was one of the channels actually used in the slaughterhouse production, with a width of 1.5 m, and all pigs passed through the channel at a normal speed (v ≤ 3 m/s). The experimental collection equipment used included a Hikvision TB-1217A-3/PA camera(manufactured by Hikvision Digital Technology Co., Ltd., Hangzhou, Zhejiang, China), at a height of 2.8 m from the ground, a video frame rate of 25 fps, and a resolution of 2688 × 1520. The data collection dates were from 1–7 February 2021, and from 25–29 March 2022, during the period of 8:00–16:00.

**Figure 2:**
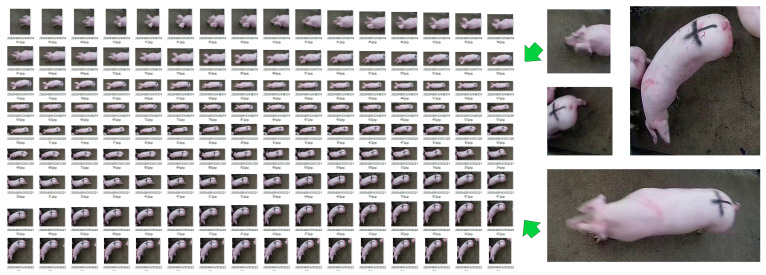
Pig target tracking dataset.

In order to implement the pig counting algorithm, we needed to create a pig detection dataset and a pig tracking dataset. Therefore, we followed the following three steps to create the datasets:

1. Effective data screening: Because pigs were not constantly passing through the channel while the videos were collected, we used VSDC Free Video Editor ×32 video editing software to clip the time periods where pigs passed through, and we manually counted the pigs in these video segments, obtaining a total of 130 video segments.
2. Pig detection dataset creation: We first used a Python 3.8 script to randomly extract 1500 .jpg format pig images from the 130 video segments, and then we manually labeled the pigs in the images using the open-source software labelImg. Finally, the dataset was divided into training, testing, and validation sets in an 8:1:1 ratio to train the YOLOv5xpig pig detection algorithm.
3. Pig tracking dataset creation: Firstly, we randomly selected 20 video segments from the 130 video segments as the pig tracking dataset. Then, to reduce the difficulty of manual data creation, we used the YOLOv5xpig pig detection algorithm to crop each pig in every frame of each video segment into a .jpg image. After manual screening and calibration, all images of the same pig were placed in the same folder, resulting in a pig tracking dataset of 197 pigs, as shown in Figure 2.

## 3. Design of MPC-YD Algorithm

Aiming to address the current difficulties faced by the YOLOv5x algorithm in detecting partial pig body parts (such as half a pig head or half a pig leg) and the problem of easy loss of pig tracking by the DeepSORT algorithm, this paper proposes an MPC-YD pig counting algorithm. The algorithm improves the detection rate of local body parts of pigs by adding two different sizes of SPP networks and using the SoftPool instead of MaxPool operation in YOLOv5x. Additionally, this algorithm includes a pig reidentification network, a spatiotemporal-state-based pig tracking correction method, and a frame-based pig counting method based on DeepSORT to improve the accuracy of pig tracking and counting.

### 3.1. MPC-YD Algorithm Architecture

**Figure 3:**
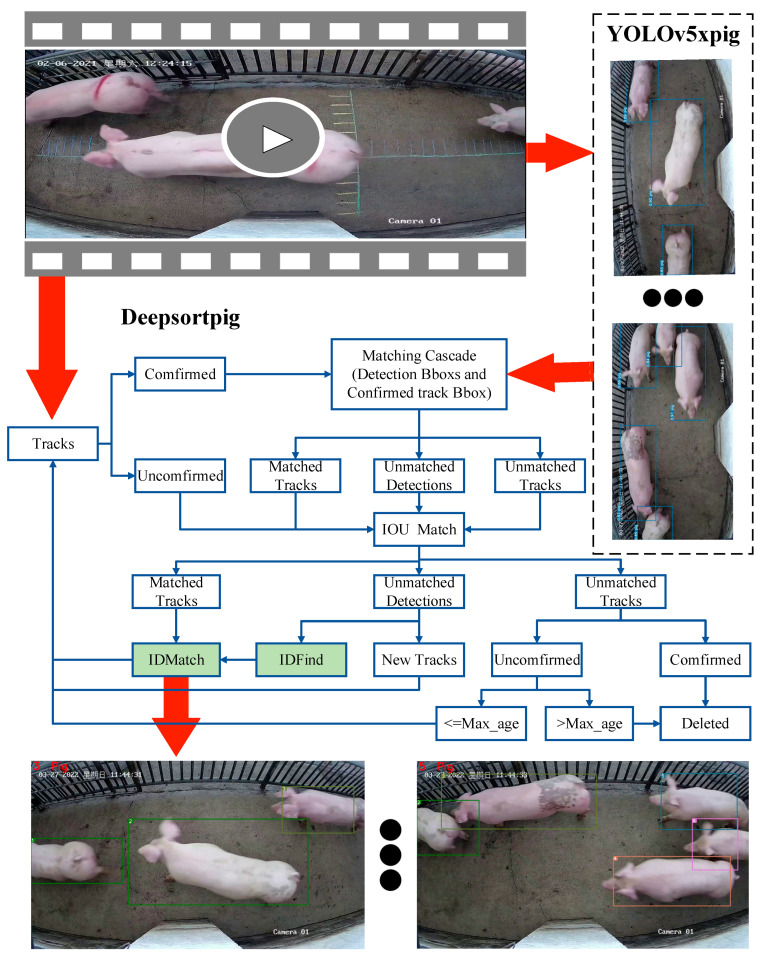
The structure of the MPC-YD(Mobile Pig Counting Algorithm with YOLOv5xpig and DeepSORTPig).

The MPC-YD pig counting algorithm is mainly divided into two parts: YOLOv5xpig pig target detection algorithm and DeepSORTpig pig target tracking and counting algorithm. The overall process is shown in Figure 3. The YOLOv5xpig pig target detection algorithm is responsible for detecting the pigs in each frame of the pig video and passing the detection results to the DeepSORTpig algorithm. After obtaining the pig target detection results in the current image, the DeepSORTpig algorithm first reads the detection results of the previous frame and predicts the pig’s motion trend in the current image. Then, the pig reidentification network is used to determine the pig association relationship between the prediction results of the previous frame and the target detection results of the current image. The ID is then assigned based on the association relationship, and the ID is corrected using a spatiotemporal-state-based pig tracking correction method (IDFind module in Figure 3). Finally, pig counting is achieved using the frame-based counting method (ID-Match module in Figure 3).

### 3.2. YOLOv5xpig Pig Counting Object Detection Algorithm

**Figure 4:**
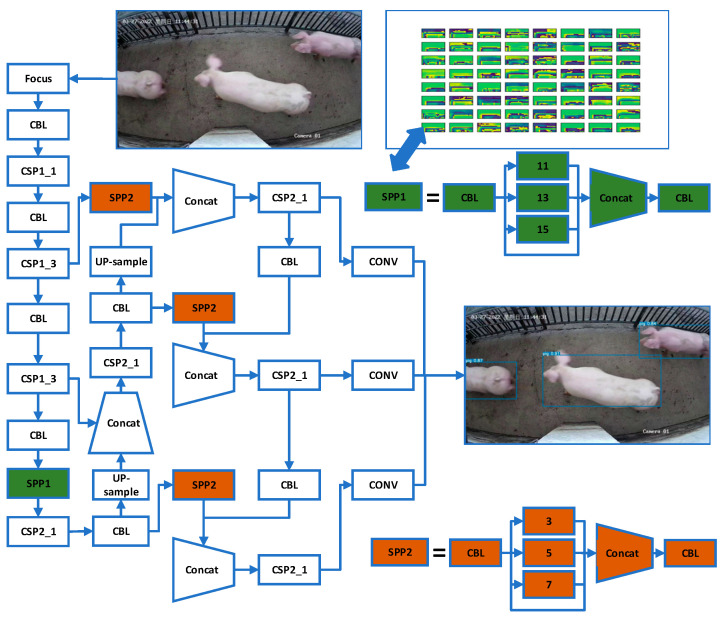
The architecture of the YOLOv5xpig algorithm includes two different SPP-net networks. YOLOv5xpig is a specific algorithm used for pig detection and recognition. SPP-net is a specific convolutional neural network structure that can handle inputs of varying sizes using spatial pyramid pooling (SPP).

The high-precision pig object detection algorithm is an important prerequisite to en-sure the accuracy of pig counting, as low-precision pig object detection algorithms can result in multiple IDs being assigned to the same pig during tracking. Considering that the YOLO model has relatively higher detection speed, is easier to deploy and apply in commercial settings than other algorithms in the field of object detection, and has more community support, we chose to adopt the YOLO model for object detection. YOLOv5 is a popular object detection network that includes four versions: YOLOv5s, YOLOv5m, YOLOv5l, and YOLOv5x. Although YOLOv5x has the lowest running speed, it has the highest detection accuracy. Because the accuracy of pig counting is more important than speed in this task, the pig object detection network YOLOv5xpig was improved based on YOLOv5x to ensure the accuracy of pig counting. During the pig counting process, it is difficult to avoid the appearance of partial pig body parts (half a pig head or half a pig leg) because all pigs exist in situations where their bodies gradually appear in the camera’s shooting range or gradually disappear from the camera’s shooting range. A spatial pyramid pooling network (SPP-net) [33] refers to a type of neural network module that is designed to address challenges related to complex shape, large size differences, and low-texture features in object detection tasks. We added two SPP-net modules with different feature block sizes to the YOLOv5x network in the YOLOv5xpig pig object detection network to improve the detection of partial pig body parts. The SPP1 feature block sizes are 1 × 1, 11 × 11, 13 × 13, and 15 × 15, while the SPP2 feature block sizes are 1 × 1, 3 × 3, 5 × 5, and 7 × 7. The improved YOLOv5xpig structure is shown in Figure 4. By using two SPP-net modules with different feature block sizes, YOLOv5xpig obtains more feature information for detection with its three different detection heads. Furthermore, to reduce the loss of feature information from partial pig body parts caused by pooling operations, YOLOv5xpig uses SoftPool [34] instead of the MaxPool operation in SPP-net, as SoftPool can better preserve detailed information in the feature map and prevent overfitting. The core idea of SoftPool is to use the SoftMax function to calculate activation weights $w_{i}$ for a feature region S with a size of C × H × W, where $a_{i}$ is the activation value in the feature region S. The formula is as follows:

(1) $$w_{i}=\frac{e^{a_{i}}}{\sum_{j\in S}e^{a_{j}}}$$

Finally, after standard summation calculation of all activation weights $w_{i}$ and activation values $a_{i}$ within feature region *S*, the output value $\tilde{a}$ of the SoftPool operation can be obtained, as shown in the following formula:

(2) $$\tilde{a}=\sum_{i\in S}w_{i}\times a_{i}$$

In addition, we used the CIOU loss to measure the loss of the bounding boxes, with a formula as follows:

(3) $${\mathrm{loss}}_{\mathrm{CIOU}}=1-\mathrm{CIOU}=1-\mathrm{IOU}+\frac{\rho^{2}}{c^{2}}+\alpha v$$

where ρ is the distance between the centers of the predicted and ground truth boxes, c is the diagonal length of the minimum bounding rectangle of the predicted and ground truth boxes, v is the normalized difference in the aspect ratio between the predicted and ground truth boxes, α is the influence factor of v, and IOU is the ratio of the intersection to the union of the predicted and ground truth boxes.

### 3.3. Pig Counting and Reidentification Network

**Figure 5:**
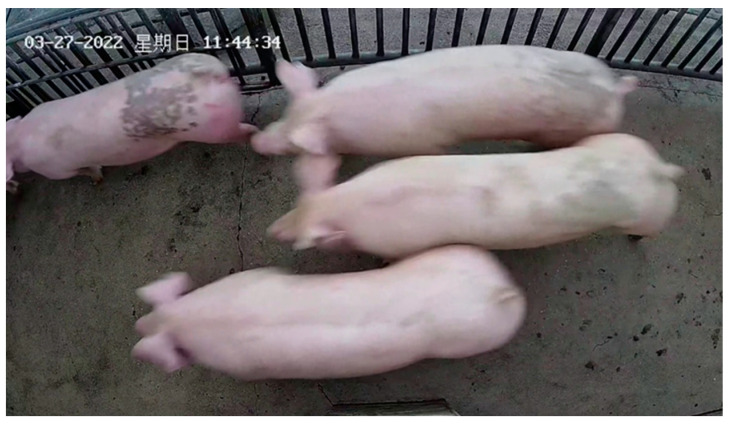
Four pigs from the same batch walking down the aisle, showing differences in pattern, coat color, and weight.

The reidentification network in the original DeepSORT algorithm was designed for pedestrians, but there are significant differences in the way pigs and humans walk. Therefore, a ratio of 1:2 is often used for pedestrian feature extraction, while a ratio of 2:1 is used for pig images to better extract pig features. Additionally, the DeepSORT algorithm uses the appearance of targets to improve tracking accuracy, which requires using a pig reidentification network to match the detected pigs in the YOLOv5xpig algorithm detection results. Although there is not much difference in appearance between pigs, in reality, there are significant differences in the characteristics among individual pigs, such as coat color, pattern, and body type, as shown in Figure 5. In order to improve the pig tracking matching rate, we designed a pig reidentification network (pig reidentification model) based on the characteristics of pig image sizes, as shown in Table 1. The pig reidentification model mainly includes 1 convolutional layer, 1 max pooling layer, 9 residual layers, and 1 average pooling layer. Finally, the output feature dimension of the pig r-identification network is increased from 128 to 512 compared with the original network.

### 3.4. Pig Tracking Method Based on Spatial State Correction

DeepSORT is an improvement upon the SORT object tracking algorithm, which defines tracking scenarios and cascade matching strategies using an eight-dimensional spatial state $\left(u,v,\gamma ,h,\dot{u},\;\dot{v},\dot{\gamma },\dot{h}\right)$ to achieve higher object tracking accuracy. Although DeepSORT has good tracking performance, rapid movements, mutual compression, or posture changes of pigs may cause the algorithm to fail in tracking them. In the task of counting pigs in corridors, new complete pigs cannot suddenly appear in the center of the video screen: they can only appear at the two ends of the screen. Therefore, if a new complete pig’s ID appears in the center of the video, the ID must be incorrect and needs to be corrected. Based on these practical situations, we designed a pig tracking algorithm based on spatial state correction on the basis of the DeepSORT algorithm. The algorithm is implemented through the IDFind module, shown in Figure 3. The specific steps are as follows:

(1) Read all pig IDs from the current and previous frames, and use the positions and IDs of pigs in both frames to identify new and lost pigs.
(2) Use the (v,γ,h) state in the eight-dimensional spatial state $\left(u,v,\gamma ,h,\dot{u},\dot{v},\dot{\gamma },\dot{h}\right)$ to determine whether a new pig ID needs to be corrected. Calculate R using Formula (4); if R > 0, then there is a complete pig that is not close to the edge of the image, and the pig ID needs to be corrected.
  (4) $$R=\left(120,000\;-\;\gamma \times h^{2}\right)\times \;v+30,000$$
  where γ is the aspect ratio of the detection box, h is the height of the detection box, and v represents the x value of the detection center $\left(x,y\right)$, 120,000 is the maximum detection area for pig images, and 30,000 is one-quarter of the maximum detection area for pig images. The maximum detection area for pig images can be obtained in Formula (5) below. Meanwhile, we think that only displaying pigs with a body volume greater than 1/4 indicates that they have entered the image. In practical use, the parameter 3000 can be adjusted based on the specific situation.
(3) Read all lost pig information, use the Euclidean distance to find the lost pig closest to the new pig, and replace the new pig ID with the old pig ID.

### 3.5. Pig Counting Method Based on Frame Number and Detection Area Judgment

When pigs stay at the edge of a video for a long time, they may have fewer features or frequently enter and exit the video edge, which makes it easy for the DeepSORT algorithm to double-count pigs at the edge. To address the issue of pigs at the video edge being prone to incorrect counting, this paper proposes a pig counting algorithm based on frame number and detection area judgment. This method is implemented through the ID Match module, as shown in Figure 3. The principle of this method is that when pigs pass through the aisle, the pixel area of their bodies gradually increases and then decreases in the video, forming a process of first increasing and then decreasing. The steps of the pig counting method based on frame number judgment are as follows:

(1) In order to record all pig information for counting calibration and judgment, a two-dimensional list List(id,n,γ,h,v) is used to save all pig information in chronological order after being corrected based on spatial state. Here, id represents the pig number, n represents the total number of times the pig has appeared in the different frames of the current video, $\left(\gamma ,h\right)$ represent the aspect ratio and detection height of the pig bounding box, and v represents the x value of the detection center $\left(x,y\right)$.
(2) The average frame number z and the maximum detection area s of pigs in the target tracking dataset are calculated through List.
(3) Formula (5) is used to determine whether the current pig needs to be counted. If N > 0, it is considered a valid count (the same pig has appeared in multiple frames with a complete body), and pig counting is performed. Then, the information of the counted pig is recorded using List1(id,n,v).
  (5) $$N=\gamma \times \;h^{2}\times \;n-z\times s\times n/2$$
  where γ is the aspect ratio of the detection box, h is the height of the detection box, n represents the total number of times the pig appears in different frames in the current video, z is the average frame number of pigs in the target tracking dataset, and s is the maximum image area of pigs in the target tracking dataset.
(4) The change process of the v value in List and List1 is used to determine whether the counted pig has turned back. If a pig disappears from view and then turns back, the counting result needs to be adjusted according to the number of times that pig has turned back to ensure counting accuracy.

## 4. Evaluation Metrics and Experimental Process

### 4.1. Experimental Environment and Training Parameters

The experiments in this study were conducted on a Windows 10 operating system with hardware configurations consisting of an Intel Core i7-9700 CPU@3.00 GHz and an NVIDIA GeForce RXT 2080Ti GPU. The main operating environment included Pytorch 1.6, Python 3.8, and CUDA 11.4. The training parameters for YOLOv5xpig and DeepSORTpig are shown in Table 2.

### 4.2. Evaluation Metrics for the MPC-YD Pig Counting Algorithm

The evaluation metrics in this study were divided into three parts: pig object detection, pig object tracking, and pig counting. For pig object detection, metrics including model size (Ms), mean average precision (including mAP:0.5 and mAP:0.95), recall (R), and FPS were used to evaluate the detection performance of the model.

(6) $$P=\frac{\mathrm{TP}}{\mathrm{TP}+\mathrm{FP}}$$

(7) $$R=\frac{\mathrm{TP}}{\mathrm{TP}+\mathrm{FN}}$$

(8) $$\mathrm{AP}=\int_{0}^{1}\;P\left(R\right)\mathrm{dR}$$

(9) $$\mathrm{mAP}=\frac{\sum_{i=1}^{c}\;\mathrm{AP}\left(i\right)}{c}$$

TP represents the number of correctly identified pigs, FP represents the number of image regions that are incorrectly identified as pigs, FN represents the number of pig regions that are incorrectly identified, and AP represents the area under the precision-recall curve (PR curve).

For pig target tracking, accuracy (Acc), multiobject tracking accuracy (MOTA), and multiobject tracking precision (MOTP) were used as indicators to evaluate the model. Acc was used to evaluate the model’s accuracy in recognizing pigs, MOTA was used to evaluate the accuracy of the tracker’s continuous tracking results, and MOTP was used to evaluate the accuracy of the tracker’s predicted target positions.

(10) $$\mathrm{Acc}=\frac{\mathrm{TP}+\mathrm{TN}}{\mathrm{TP}+\mathrm{TN}+\mathrm{FP}+\mathrm{FN}}$$

(11) $$\mathrm{MOTA}=1-\frac{\sum_{t}\;\left(m_{t}+fp_{t}+{\mathrm{mme}}_{t}\right)}{\sum_{t}\;g_{t}}$$

(12) $$\mathrm{MOTP}=\frac{\sum_{i,t}\;d_{t}^{i}}{\sum_{t}\;c_{t}}$$

In Equation (10), TP represents the number of correctly identified pigs, FP represents the number of image regions that are incorrectly identified as pigs, TN represents the number of correctly identified nonpig regions, and FN represents the number of pig regions that are incorrectly identified. In Equation (11), t represents the tth frame of the image, $m_{t}$ represents the number of missed detections in the tth frame, $fp_{t}$ represents the number of false positives in the tth frame, $mme_{t}$ represents the number of mismatches in the tth frame, and $g_{t}$ represents the number of correct detections in the tth frame. In Equation (12), i refers to the matching result number, $d_{t}^{i}$ represents the distance error between the matched target in the tth frame image and the predicted correct position, and $c_{t}$ represents the number of matches in the tth frame image.

For pig counting results, mean absolute error (MAE) and coefficient of determination ($R^{2}$) were used as indicators to evaluate the accuracy of the counting. The relevant calculation formulas are as follows:

(13) $$\mathrm{MAE}=\frac{1}{n}\sum_{i=1}^{n}\;\left|{\hat{y}}_{i}-y_{i}\right|$$

(14) $$R^{2}=1\;-\;\frac{\sum_{i=1}^{n}\;{\left(y_{i}-{\hat{y}}_{i}\right)}^{2}}{\sum_{i=1}^{n}\;{\left(y_{i}-\overset{-}{y}\right)}^{2}}$$

where $y_{i}$ represents the true value, ${\hat{y}}_{i}$ represents the predicted value, and $\overset{-}{y}$ represents the mean of all true values.

### 4.3. Training of the MPC-YD Pig Counting Algorithm

**Figure 6:**
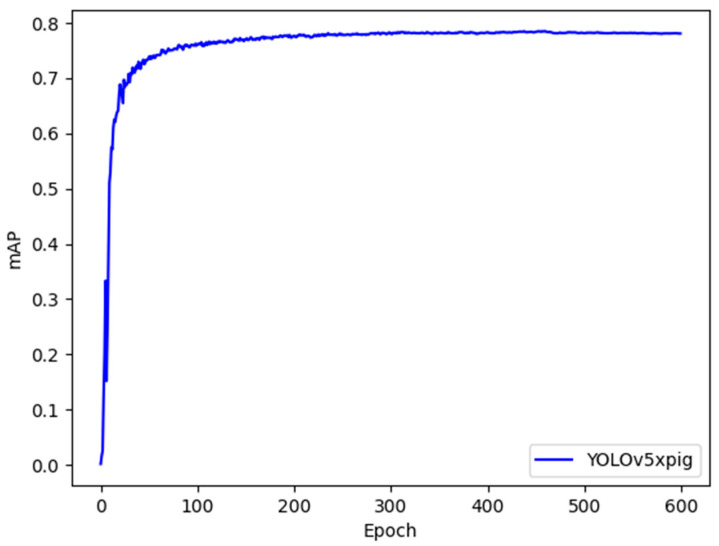
Training curve of YOLOv5xpig with mAP of 0.95.

The training curve of YOLOv5xpig with mAP of 0.95 is shown in Figure 6. From the graph, it can be observed that the model detection accuracy rapidly improves before 100 iterations, while the improvement rate gradually slows down between 100 and 200 iterations and starts to converge. Finally, at 500 iterations, the model detection accuracy tends to stabilize, and the model achieves the desired performance.

**Figure 7:**
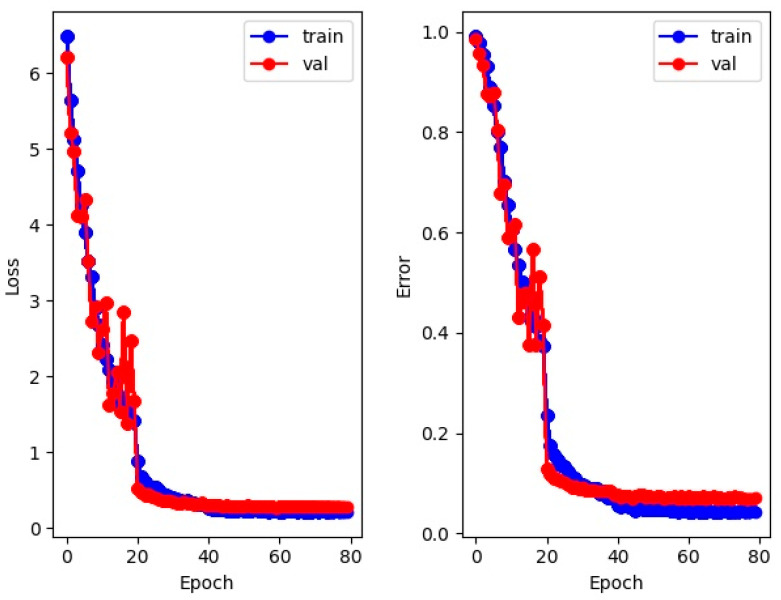
Training of the pig reidentification network, including the loss curve and error curve training results.

The training effect of the DeepSORTpig algorithm on pig reidentification using the pig target tracking dataset is shown in Figure 7. From the graph, it can be observed that the model accuracy rapidly decreases before 20 iterations but with large fluctuations. This is because there are significant differences in image size, shape, and pig posture in the pig target tracking dataset (Figure 2). From the loss curve and A curve, it can be seen that the model basically converges after 40 iterations; at this point, the model accuracy (ACC) reached 95.32%, indicating that it can accurately extract pigs’ phenotype features.

## 5. Analysis of Results

### 5.1. Analysis of Pig Object Detection Accuracy

In order to analyze the performance of the pig object detection algorithm, we compared YOLOv5xpig with other networks (YOLOv5s, YOLOv5m, YOLOv5l, YOLOv5x, and faster R-CNN), as shown in Table 3. From Table 3, it can be seen that although the YOLOv5xpig model is larger, its average precision (mAP:0.5 and mAP:0.95) is higher than that of YOLOv5s, YOLOv5m, YOLOv5l, YOLOv5x, and faster R-CNN. Moreover, the average precision (mAP:0.95) of YOLOv5xpig is 7.07% higher than that of YOLOv5x, and the average precision (mAP:0.5) is 3.12% higher than that of YOLOv5x. In addition, although the 33 FPS of YOLOv5xpig is relative low, it is nevertheless higher than the 25 FPS accuracy of the camera used in this study, which fully meets production requirements.

**Figure 8:**
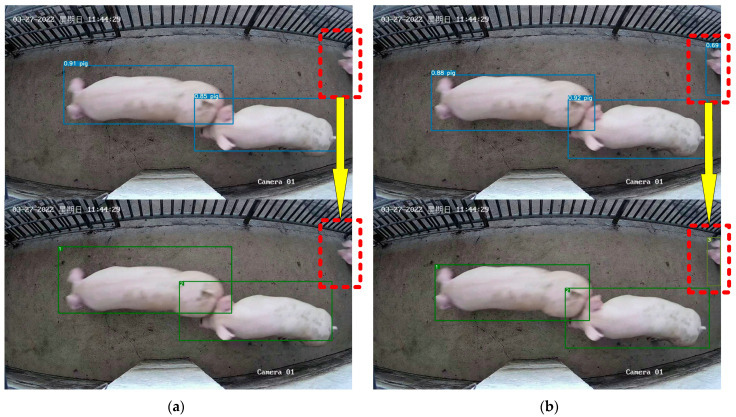
Analysis of detection and tracking results of YOLOv5x and YOLOv5xpigs: (**a**) YOLOv5x + DeepSORT cannot recognize pig No. 3; (**b**) YOLOv5xpig + DeepSORT can recognize pig No. 3. The red dashed box in the figure represents the results of object detection and tracking, while the yellow arrows indicate different object tracking results caused by different object detection results.

The YOLOv5xpig algorithm can better detect local body parts of pigs than YOLOv5x, as shown in Figure 8. From Figure 8, it can be seen that YOLOv5xpig can detect piglet heads, which YOLOv5x cannot. Furthermore, because YOLOv5x cannot detect the piglet heads, as shown in Figure 8, it cannot identify pig No. 3 in the video, but it can be successfully tracked using YOLOv5xpig. Therefore, Figure 8 shows that the YOLOv5xpig algorithm can enable the MPC-YD algorithm to track pigs with only local body features, indicating that improving the detection of local body parts of pigs is very meaningful for improving pig tracking performance.

### 5.2. Analysis of Pig Object Tracking Accuracy

In order to verify the improved DeepSORT algorithm’s performance in pig tracking, we compared the improved DeepSORT algorithm with the original algorithm, as shown in Table 4. From Table 4, it can be seen that the MOTA of the improved DeepSORT algorithm was improved by 1.2% compared with the original algorithm, but the MOTP was only improved by 0.34%. This is because the pig tracking method based on spatial state correction added in this study has the function of reducing the probability of target loss during pig tracking, thus improving the continuity of pig tracking, resulting in a greater improvement in MOTA.

**Figure 9:**
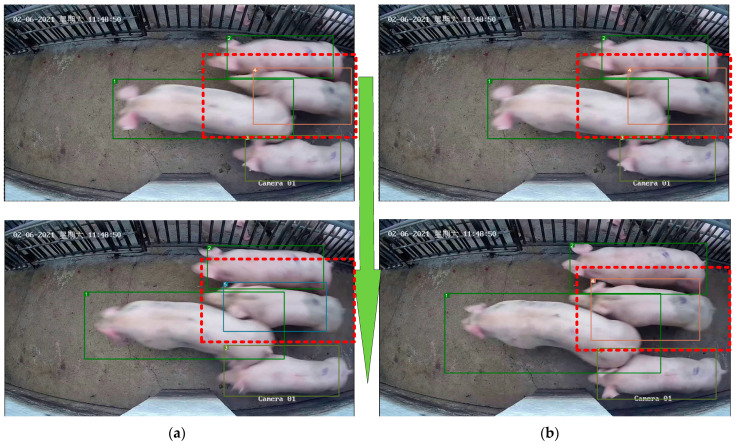
Comparison of DeepSORT algorithm before and after adding the pig tracking method based on spatial state correction. (**a**) DeepSORT mistakenly tracked pig No. 5 instead of pig No. 4. (**b**) DeepSORTpig correctly tracked pig No. 4. The red dashed box in the figure presents the tracked target that requires particular attention, while the green arrows show the variations between the two consecutive frames from top to bottom.

The comparison of the DeepSORTpig algorithm with and without the pig tracking method based on spatial state correction is shown in Figure 9. From Figure 9, it can be seen that the original algorithm lost track of pig No. 4 due to its rapid passage through the channel, resulting in pig No. 4 being misidentified as pig No. 5. However, according to the rules of the pig tracking method based on spatial state correction, if the bounding box area of pig No. 4 in the previous frame is large and the center of the bounding box is not on the edge of the image, it can be considered that pig No. 4 has not disappeared in the current image. Then, the tracking of pig No. 4 can be corrected based on the detection box and ID assignment. Finally, pig No. 4 is successfully maintained in the correct tracking state.

**Figure 10:**
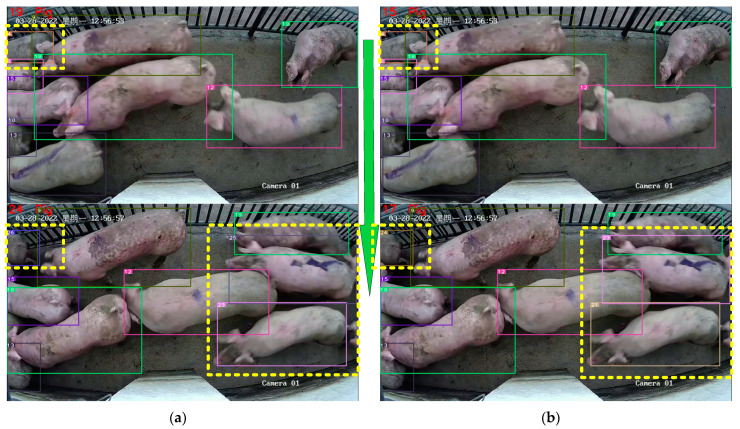
Comparison of pig counting results before and after adding the pig counting method based on frame number judgment. (**a**) The counting result in DeepSORT changed from 19 to 24. (**b**) The counting result in DeepSORTpig algorithm changed from 15 to 17, and this result is correct. The yellow dashed box in the figure presents the pig ID that requires particular attention, while the green arrows show the variations between the two consecutive frames from top to bottom.

Although the pig counting method based on frame number cannot improve the accuracy of pig tracking by DeepSORTpig, it can improve the accuracy of pig counting, as shown in Figure 10. Due to the busy state of the slaughterhouse, pig No. 4, shown in Figure 10a, remained at the edge of the video and was constantly squeezed by other pigs, causing its ID to change from 4 to 26. This also resulted in the original counting algorithm’s count changing from 19 to 24, while the actual correct count should have changed from 15 to 17. However, by introducing a pig counting algorithm based on the number of frames and detection area, as shown in Figure 10b, even though the ID of pig No. 4 changed from 4 to 24, this algorithm only counted pigs No. 21 and No. 23, resulting in the correct count of 17. This successfully prevented the occurrence of duplicate pig counting due to pig ID changes caused by squeezing and prolonged stay.

### 5.3. Analysis of Pig Counting Results

**Figure 11:**
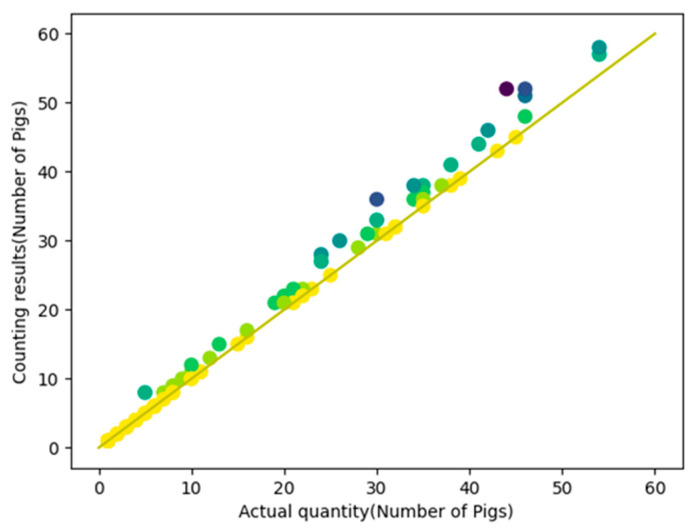
Pig counting two-dimensional image result. The yellow dots represent the counting results that match the ground truth, while darker dots indicate larger discrepancies between the counting results and the ground truth.

**Figure 12:**
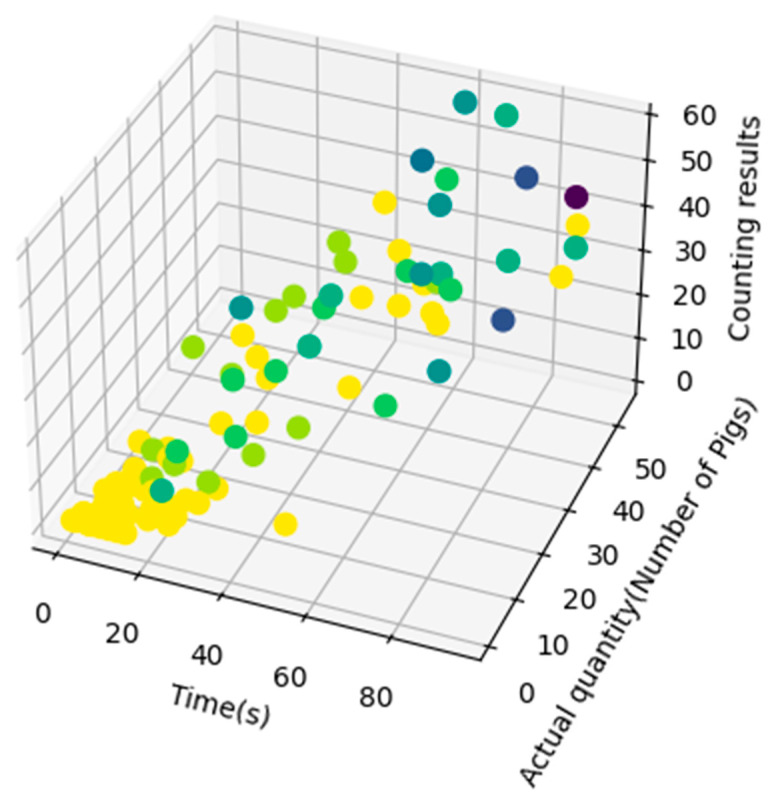
Pig counting three-dimensional image result. The yellow dots represent the counting results that match the ground truth, while darker dots indicate larger discrepancies between the counting results and the ground truth.

In order to verify the counting accuracy in this experiment, a total of 1695 pigs in 94 videos were counted, producing an MAE of 1.03 and an R^2^ of 98.39%, as shown in Figure 11. From Figure 11, it can be seen that the error probability of the pig counting algorithm increases with the increased in the number of pigs, but the maximum error did not exceed eight pigs. This is because as the number of pigs increases, the situation of crowding between pigs becomes more complex. At the same time, a 3D scatter plot of pig counting was established using the pig video time, actual number, and algorithmic counting results, as shown in Figure 12. From Figure 12, it can be seen that the probability of counting errors increases with increases in pig numbers and video duration. Moreover, when the video duration is short but the number of pigs is large, errors more easily occur, as crowding at the channel exits is also more likely to occur, which increases the complexity of pig tracking due to the large number of pigs squeezing each other. However, considering Figure 11 and Figure 12, it can be seen that when the number of pigs is no more than 10, the algorithm produced only 4 videos out of 41 with counting errors, with an MAE of 0.14. The error values of these four videos were one, one, one, and three, which meet the requirements for verifying the accuracy of manual counting for small slaughterhouses.

### 5.4. Counting Test in Different Breeding Environments

**Figure 13:**
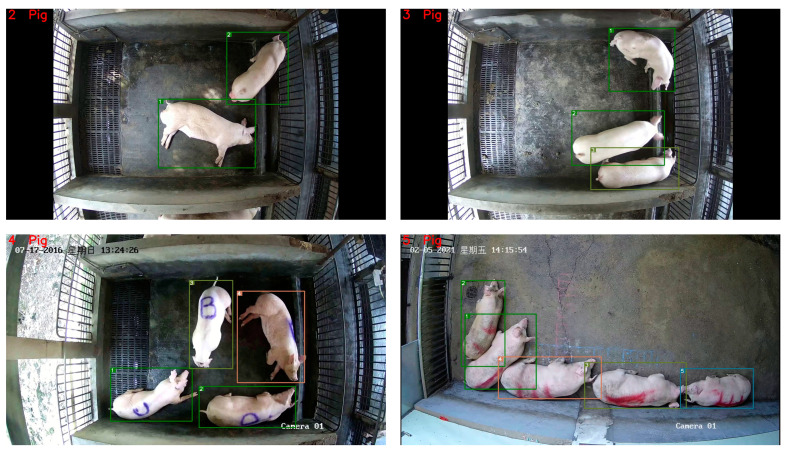
Pig counting result in breeding environment.

In order to verify the generalization of the counting algorithm, we tested pig breeding videos with different numbers of pigs for a duration of 30 s, as shown in Figure 13. From Figure 13, it can be seen that the algorithm correctly counted two, three, four, and five pigs, indicating that the algorithm can be used for counting in conventional breeding environments and has good generalization. In addition, because pigs in breeding environments can appear in the video for a long time, which is different from the situation in the aisle of a slaughterhouse, it was necessary to set the MAX_AGE parameter to not execute the ID deletion operation and reset the MAX_AGE parameter to zero when triggered, in order to prevent pig tracking from being lost due to triggering of the MAX_AGE parameter when the pig reaches the maximum frame number during counting in breeding environments.

## 6. Discussion

Although the MAE of the pig counting algorithm in this paper could reach 0.14 when the number of pigs was no more than 10, the application and promotion of this algorithm still has a lot of room for improvement, as the number of pigs in each batch entering the slaughterhouse cannot be fixed to below 10. Currently, the strategy for promoting the application of this algorithm is to split large groups of pigs into multiple groups with less than 10 pigs for multiple counting sessions, which ensures the accuracy of the pig counting. The counting effect of this algorithm under a smooth channel is shown in Video S1. In addition, when counting in a farming environment, the counting range of this algorithm is limited to no more than five pigs. However, if the number of pigs exceeds five, the small size of the farming troughs may result in a higher probability of counting errors due to pigs blocking each other while eating. Nevertheless, despite this limitation, the algorithm still possesses the ability to stably track pigs, providing important technical support for studying pig behavior patterns in farming environments. Therefore, in addition to improving the existing short-comings, the next step to improve this algorithm is to analyze pig behavior patterns by tracking them.

## 7. Conclusions

To solve the problem of low efficiency in manual pig counting and easy loss of pig tracking in video, we developed an MPC-YD pig tracking and counting method. In this algorithm, we introduced a method of embedding two SPP-nets with different feature block sizes into the YOLOv5x network to improve pig target detection accuracy, addressing the problem of difficult detection of local pig body parts. Furthermore, we added a pig reidentification network, a pig tracking method based on spatial state correction, and a pig counting method based on frame number judgment to the DeepSORT algorithm to reduce pig counting errors. Finally, the pig counting coefficient of determination (R2) of this model reached 98.14%, and it also demonstrated good tracking and counting effects in breeding environments.

It is worth mentioning that all the data used for training and validation in this method were collected from real production environments, and only one camera needs to be installed above the pig pen aisle to implement this method. Therefore, this method is easy to promote and apply and can provide technical support for intelligent pig counting in both breeding and slaughterhouse environments.

## Figures and Tables

**Table 1:** Pig reidentification network architecture.

Name	Patch Size/Stride	Output Size
Conv 1	3 × 3/1	32 × 128 × 64
Max pool 2	3 × 3/2	32 × 64 × 32
Residual 3	3 × 3/1	32 × 64 × 32
Residual 4	3 × 3/2	64 × 32 × 16
Residual 5	3 × 3/1	64 × 32 × 16
Residual 6	3 × 3/1	64 × 32 × 16
Residual 7	3 × 3/2	128 × 16 × 8
Residual 8	3 × 3/1	128 × 16 × 8
Residual 9	3 × 3/2	256 × 8 × 4
Residual 10	3 × 3/1	256 × 8 × 4
Residual 11	3 × 3/2	512 × 4 × 1
Avgpool 12	4 × 1/1	512 × 1 × 1

**Table 2:** Training parameters for YOLOv5xpig and DeepSORTpig.

YOLOv5xpig		DeepSORTpig	
Parameter Name	Parameter Value	Parameter Name	Parameter Value
Epochs	450	Epochs	80
Img-size	640	Max_DIST	0.1
Max-det	20	MIN_CONFIDENCE	0.5
Iou-thres	0.45	NMS_MAX_OVERLAP	0.6
Batchsize	2	MAX_IOU_DISTANCE	0.5

**Table 3:** Comparison of performance of different object detection models.

Task Model	MS (MB)	mAP:0.5 (%)	mAP:0.95 (%)	Recall(%)	FPS(f/s)
YOLOv5s	14.4	94.25	65.83	93.71	87
YOLOv5m	41.9	95.05	69.13	95.25	69
YOLOv5l	91.6	94.74	71.06	94.76	43
YOLOv5x	170.1	96.12	71.22	94.88	35
YOLOv5xpig	170.2	99.24	78.29	97.37	33
Faster R-CNN	360.1	94.64	68.25	88.94	19

**Table 4:** Comparison of model performance of DeepSORT before and after improvement.

Model	Detector	MOTA	MOTP
DeepSORT	YOLOv5xpig	84.12	83.79
DeepSORTpig	YOLOv5xpig	85.32	84.13

## Data Availability

The data presented in this study are available on request from the corresponding author. The data are not publicly available due to the fact that the data would reveal the operation of the slaugh-terhouse.

## Supplementary Materials

The following supporting information can be downloaded at: https://www.mdpi.com/article/10.3390/s23146309/s1, Video S1: 8 pigs counting.

## References

[1] Tian M., Guo H., Chen H., Wang Q., Long C., Ma Y. (2019). Automated pig counting using deep learning. Comput. Electron. Agric..

[2] Girshick R., Donahue J., Darrell T., Malik J. Rich Feature Hierarchies for Accurate Object Detection and Semantic Segmentation. Proceedings of the IEEE Conference on Computer Vision and Pattern Recognition (CVPR).

[3] Redmon J., Divvala S., Girshick R., Farhadi A. (2016). You Only Look Once: Unified, Real-Time Object Detection. Computer Vision & Pattern Recognition. arXiv.

[4] Ren S., He K., Girshick R., Sun J. (2017). Faster R-CNN: Towards Real-Time Object Detection with Region Proposal Networks. IEEE Trans. Pattern Anal. Mach. Intell..

[5] He K., Gkioxari G., Dollár P., Girshick R. (2017). Mask R-CNN. IEEE Trans. Pattern Anal. Mach. Intell..

[6] Huang Z., Huang L., Gong Y., Huang C., Wang X. (2019). Mask Scoring R-CNN. arXiv.

[7] Qi L., Zhang X., Chen Y., Chen Y., Jia J. (2020). PointINS: Point-based Instance Segmentation. arXiv.

[8] Bewley A., Ge Z., Ott L., Ramos F., Upcroft B. (2016). Simple Online and Realtime Tracking. arXiv.

[9] Kim C., Li F., Ciptadi A., Rehg J.M. Multiple Hypothesis Tracking Revisited. Proceedings of the 2015 IEEE International Conference on Computer Vision (ICCV).

[10] Wojke N., Bewley A., Paulus D. (2017). Simple Online and Realtime Tracking with a Deep Association Metric. arXiv.

[11] Li G., Huang Y., Chen Z., Chesser G.D., Purswell J.L., Linhoss J., Zhao Y. (2021). Practices and Applications of Convolutional Neural Network-Based Computer Vision Systems in Animal Farming: A Review. Sensors.

[12] Li G., Shi G., Jiao J. (2023). YOLOv5-KCB: A New Method for Individual Pig Detection Using Optimized K-Means, CA Attention Mechanism and a Bi-Directional Feature Pyramid Network. Sensors.

[13] Dong S., Wang R., Liu K., Jiao L., Li R., Du J., Teng Y., Wang F. (2021). CRA-Net: A channel recalibration feature pyramid network for detecting small pests. Comput. Electron. Agric..

[14] Li D., Chen Y., Zhang K., Li Z. (2019). Mounting Behaviour Recognition for Pigs Based on Deep Learning. Sensors.

[15] Tu S., Yuan W., Liang Y., Wang F., Wan H. (2021). Automatic Detection and Segmentation for Group-Housed Pigs Based on PigMS R-CNN. Sensors.

[16] Zhou H., Li Q., Xie Q. (2023). Individual Pig Identification Using Back Surface Point Clouds in 3D Vision. Sensors.

[17] Brunet H., Creach P., Concordet D. (2023). Optimal estimation of broiler movement for commercial tracking. Smart Agric. Technol..

[18] Bellocchio E., Crocetti F., Costante G., Fravolini M.L., Valigi P. (2022). A novel vision-based weakly supervised framework for autonomous yield estimation in agricultural applications. Eng. Appl. Artif. Intell..

[19] Liu Z., Wang Q., Meng F. (2022). A benchmark for multi-class object counting and size estimation using deep convolutional neural networks. Eng. Appl. Artif. Intell..

[20] Lins E.A., Rodriguez J.P.M., Scoloski S.I., Pivato J., Lima M.B., Fernandes J.M.C., Da Silva Pereira P.R.V., Lau D., Rieder R. (2020). A method for counting and classifying aphids using computer vision. Comput. Electron. Agric..

[21] Zhao Y., Wu W., Zhou Y., Zhu B., Yang T., Yao Z., Ju C., Sun C., Liu T. (2022). A backlight and deep learning based method for calculating the number of seeds per silique. Biosyst. Eng..

[22] Gao F., Fang W., Sun X., Wu Z., Zhao G., Li G., Li R., Fu L., Zhang Q. (2022). A novel apple fruit detection and counting methodology based on deep learning and trunk tracking in modern orchard. Comput. Electron. Agric..

[23] Kestur R., Meduri A., Narasipura O. (2019). MangoNet: A deep semantic segmentation architecture for a method to detect and count mangoes in an open orchard. Eng. Appl. Artif. Intell..

[24] Kim J., Suh Y., Lee J., Chae H., Ahn H., Chung Y., Park D. (2022). EmbeddedPigCount: Pig Counting with Video Object Detection and Tracking on an Embedded Board. Sensors.

[25] Oczak M., Maschat K., Berckmans D., Vranken E., Baumgartner J. (2016). Automatic estimation of number of piglets in a pen during farrowing, using image analysis. Biosyst. Eng..

[26] Huang E., Mao A., Gan H., Camila Ceballos M., Parsons T.D., Xue Y., Liu K. (2021). Center clustering network improves piglet counting under occlusion. Comput. Electron. Agric..

[27] Jensen D.B., Pedersen L.J. (2021). Automatic counting and positioning of slaughter pigs within the pen using a convolutional neural network and video images. Comput. Electron. Agric..

[28] Chen G., Shen S., Wen L., Luo S., Bo L. (2020). Efficient Pig Counting in Crowds with Keypoints Tracking and Spatial-aware Temporal Response Filtering. arXiv.

[29] Wu B., Liu C., Jiang F., Li J., Yang Z. (2023). Dynamic identification and automatic counting of the number of passing fish species based on the improved DeepSORT algorithm. Front. Environ. Sci..

[30] Cao Y., Chen J., Zhang Z. (2023). A sheep dynamic counting scheme based on the fusion between an improved-sparrow-search YOLOv5x-ECA model and few-shot deepsort algorithm. Comput. Electron. Agric..

[31] Chen X., Pu H., He Y., Lai M., Zhang D., Chen J., Pu H. (2023). An Efficient Method for Monitoring Birds Based on Object Detection and Multi-Object Tracking Networks. Animals.

[32] Wojke N., Bewley A. Deep Cosine Metric Learning for Person Re-identification. Proceedings of the 2018 IEEE Winter Conference on Applications of Computer Vision (WACV).

[33] He K., Zhang X., Ren S., Sun J. (2015). Spatial Pyramid Pooling in Deep Convolutional Networks for Visual Recognition. IEEE Trans. Pattern Anal. Mach. Intell..

[34] Stergiou A., Poppe R., Kalliatakis G. Refining activation downsampling with SoftPool. Proceedings of the IEEE/CVF International Conference on Computer Vision (ICCV).

